# Optimization, Characterization and Pharmacokinetic Study of Meso-Tetraphenylporphyrin Metal Complex-Loaded PLGA Nanoparticles

**DOI:** 10.3390/ijms222212261

**Published:** 2021-11-12

**Authors:** Mariia R. Mollaeva, Nikita Yabbarov, Maria Sokol, Margarita Chirkina, Murad D. Mollaev, Artur Zabolotskii, Irina Seregina, Mikhail Bolshov, Alexander Kaplun, Elena Nikolskaya

**Affiliations:** 1 N.M. Emanuel Institute of Biochemical Physics of Russian Academy of Sciences, 119991 Moscow, Russia; yabbarovng@gmail.com (N.Y.); mariyabsokol@gmail.com (M.S.); fom.marg@mail.ru (M.C.); 2 JSC Russian Research Center for Molecular Diagnostics and Therapy, 117149 Moscow, Russia; md-mollaev@yandex.ru (M.D.M.); zabolotscky.artur@yandex.ru (A.Z.); 3 Dmitry Rogachev National Medical Research Center of Pediatric Hematology, Oncology and Immunology, 117198 Moscow, Russia; 4 Chemistry Department, Lomonosov Moscow State University, 119234 Moscow, Russia; sereginairinaf@mail.ru (I.S.); mbolshov@mail.ru (M.B.); 5 Lomonosov Institute of Fine Chemical Technologies, MIREA—Russian Technological University, 119454 Moscow, Russia; alexander.p.kaplun@gmail.com

**Keywords:** nanoparticles, PLGA, metalloporphyrins, Box–Behnken design, drug release, kinetics, binding constant, hemolytic activity, pharmacokinetics

## Abstract

The selection of technological parameters for nanoparticle formulation represents a complicated development phase. Therefore, the statistical analysis based on Box–Behnken methodology is widely used to optimize technological processes, including poly(lactic-co-glycolic acid) nanoparticle formulation. In this study, we applied a two-level three-factor design to optimize the preparation of nanoparticles loaded with cobalt (CoTPP), manganese (MnClTPP), and nickel (NiTPP) metalloporphyrins (MeP). The resulting nanoparticles were examined by dynamic light scattering, X-ray diffraction, Fourier transform infrared spectroscopy, MTT test, and hemolytic activity assay. The optimized model of nanoparticle formulation was validated, and the obtained nanoparticles possessed a spherical shape and physicochemical characteristics enabling them to deliver MeP in cancer cells. In vitro hemolysis assay revealed high safety of the formulated MeP-loaded nanoparticles. The MeP release demonstrated a biphasic profile and release mechanism via Fick diffusion, according to release exponent values. Formulated MeP-loaded nanoparticles revealed significant antitumor activity and ability to generate reactive oxygen species. MnClTPP- and CoTPP-nanoparticles specifically accumulated in tissues, preventing wide tissue distribution caused by long-term circulation of the hydrophobic drug. Our results suggest that MnClTPP- and CoTPP-nanoparticles represent the greatest potential for utilization in in anticancer therapy due to their effectiveness and safety.

## 1. Introduction

The most widely known metalloporphyrins (MeP) are natural heme (Fe containing) and chlorophyll (Mg containing) [1,2]. These MeP are characterized by high catalytic activity, which allows their engagement in respiration and photosynthesis processes [3,4,5,6,7,8,9]. MeP catalytic activity determines their widespread application in industry and medicine (optical and contrast imaging, photosensitizers, theranostics, solar cells, etc.) [10,11,12,13,14,15,16,17,18,19,20,21,22]. Mn-based porphyrin derivatives are being tested as a radioprotector in phase II clinical trials in the USA [12,13,14]. In addition to the application in chemotherapy, MeP are also practiced in photodynamic therapy (PDT) as photosensitizers [15,16,17,18]. Another promising method for cancer treatment using MeP is a catalyst therapy, based on the interaction of MeP and substrate (e.g., ascorbic acid (AA)) [19]. During redox reaction, the formation of reactive oxygen species (ROS) occurs, provoking tumor cell death [23]. Currently, cationic Mn (III) N-substituted pyridyl- and N,N′-disubstituted imidazolyl-porphyrins are tested as catalyst therapy drugs [24,25,26,27,28]. Thus, MeP represent promising agents for cancer treatment. However, MeP application is often limited due to their low water solubility. MeP encapsulation into polymer nanocarriers is one of the methods for increasing its bioavailability [29,30,31].

**Figure 1:**
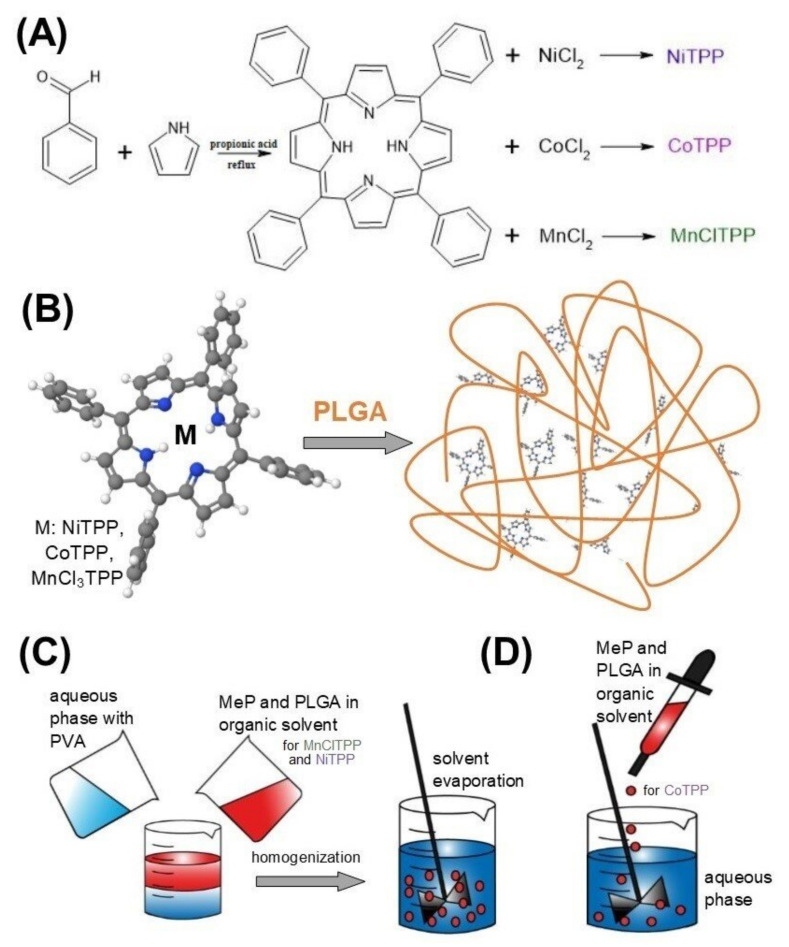
Synthesis of the MeP (**A**), preparation scheme of MeP-loaded PLGA NPs (**B**), formulation of NiTPP- and MnClTPP-loaded NPs using the single emulsion-solvent evaporation method (**C**) and the nanoprecipitation method for the preparation of CoTPP-NPs (**D**).

In recent years, the progress in targeted delivery systems has allowed biological barriers to be overcome and eliminated the disadvantages of both new drugs and approval, significantly increasing their effectiveness. Thus, lipophilic drug encapsulation into a polymer may solve the problem of solubility and reduce side effects (Figure 1B). Recent examples demonstrated the successful application of nanotechnologies in the development of delivery systems with controlled and targeted drug release [32]. Nanoparticles (NPs) are of great interest in medicine as drug delivery systems, since they are able to move freely and stably in the bloodstream compared to larger particles. NPs possess unique structural, chemical, mechanical, magnetic, electrical, and biological properties [32]. The physicochemical properties of NPs and the pathophysiological properties of the tumor microenvironment influence the drug toxicity and pharmacokinetics. The size and charge of NPs are critical parameters affecting their accumulation in the tissue of interest. Particle size plays a crucial role in the enhanced permeability and retention effect in tumor tissues, since the pore size of tumor vessels is in the range from 200 to 800 nm [33]. Moreover, the particle size must be more than 6 nm to escape kidney excretion or less than 500 nm to avoid internalization by macrophages [34]. Currently, many different types of nanocarriers are used in nanotechnology (liposomes, solid-lipid NPs, nanogels, silver/gold NPs, dendrimers) [35,36,37,38,39,40]. Polymer NPs play an important role in optimization of tumor-targeted drug delivery due to their controlled particle size, high drug loading, relative monodispersity, and adjustable drug release. Poly(lactic-co-glycolic acid) (PLGA) is a well-known polymer used in drug delivery. The main advantages of PLGA include its biocompatibility, biodegradability, and mechanical strength [37,41].

Formulation factors (rotation speed, homogenization time, surfactant and organic solvent concentration, etc.) influence the optimization method of NPs preparation, chemical properties of NPs, and drug release [41,42]. Hence, during the optimization process, it is necessary to vary these factors between extremum values. Concerning this, the Box–Behnken experimental design (BBD) is used. The advantage of BBD is a lack of combinations where factors are simultaneously at high or low levels as well as a decreased number of experiments [43]. Therefore, the BBD method is widely used for optimization of NP preparation [43,44,45].

In this research, we encapsulated MnCl_3_-tetraphenylporphyrin chloride (MnClTPP), Ni^II^-, and Co^II^-tetraphenylporphyrins (NiTPP and CoTPP) in PLGA NPs. We applied the Box–Behnken experimental design to investigate the effect of formulation parameters on the MeP loading content (LC) and particle size (PS). After NPs optimization, we analyzed their chemical properties, rates and release patterns, in vitro hemolysis ability, antitumor activity, and ROS generation potential. We also compared the pharmacokinetic profile after intravenous administration in animal models with the results of MeP release and binding constant in vitro.

## 2. Results and Discussion

### 2.1. Optimization of Process Parameters for NP Preparation

Nanoprecipitation and single emulsion-solvent evaporation are among the most popular methods of polymer particle synthesis due to their simplicity and reproducibility. During the NP formulation, many parameters may be varied to optimize the characteristics of the final product. In the present research, we applied BBD in the optimization of the formulation of MeP-loaded PLGA NPs. We describe the optimization of the single emulsion-solvent evaporation method in detail only in the NiTPP case for brevity. In the MnClTPP and CoTPP cases, the calculated and experimental data are fully represented in the Supplementary Materials and briefly described below (Supplementary Materials). The high LC and low PS were considered as essential parameters during the experiment design. Preliminary experiments allowed us to select an appropriate NP preparation method and evaluate the influence of various factors on the characteristics of the formulated NPs [46,47]. We designed 17 experiments with five center points wherein three parameters varied: amount of PLGA (mg), PVA concentration (%, *w*/*v*), and organic to aqueous phase ratio (*v*/*v*), while other process parameters remained unchanged (Table 1).

**Figure 2:**
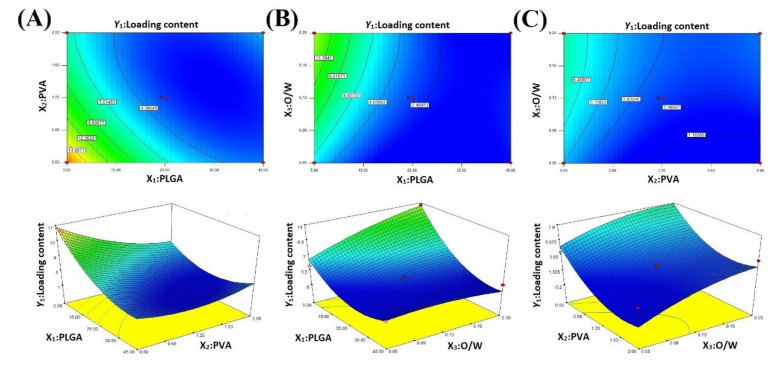
Response surface plots of the effects on NiTPP LC (Y_1_) of (**A**) the PLGA amount and PVA concentration; (**B**) PVA concentration and organic/aqueous phase ratio; and (**C**) PLGA amount and organic/aqueous phase ratio.

**Figure 3:**
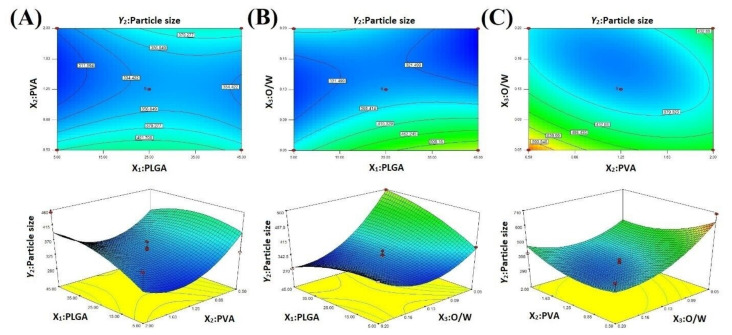
Response surface plots showing the effects on NiTPP PS of (**A**) PLGA mass and PVA concentration; (**B**) PVA concentration and organic/aqueous phase ratio; (**C**) PLGA and organic/aqueous phase ratio.

PS and NiTPP LC were the essential characteristics of the NPs formulated. We applied BBD to optimize MeP-loaded NP preparation: two- and three-dimensional diagrams of response surfaces interpret the effects of independent factors on response functions. These diagrams allowed us to evaluate the influence of the most significant factors on the NP characteristics (Figure 2 and Figure 3). The statistical analysis revealed that the mathematical model fitted the data and analysis performed using ANOVA. The model was statistically significant (*p* < 0.05) with a minor lack of fit (*p* > 0.05) for all chosen responses.

We tested the selected response surfaces against various mathematical models: linear (first order), 2FI (two factor interaction), quadratic (second order), and cubic. The significance of the *p*-value and *F*-value (highest values) of the standard deviation (less than 0.05) suggested that the quadratic model was more appropriate for optimization of the NP formulation (Table 2). We confirmed the model adequacy for the dependent parameter (LC) using the *p*-value (0.034 < 0.05) and determination coefficient (R^2^) (0.9049 > 0.9000). The PS values were close to the comparison numbers (*p*-value: 0.0518 > 0.0500, R^2^: 0.8258 < 0.9000), which partially questioned the adequacy of the prediction model for this parameter. ANOVA showed that the quadratic model is reliable and accurate towards pure error. The input parameters X_1_, X_1_^2^ in the analysis of LC and X_3_, X_3_^2^ in the analysis of PS were the most significant.

### 2.2. Loading Content

LC is an essential characteristic of NPs, because the successful treatment with therapeutic NPs is closely associated with LC affecting drug toxicity and administration frequency. LC of NiTPP-NPs was found in the range of 1.9% to 19.9% (Table 1). During the formulation process, we varied the following factors: PLGA ratio (mg) (X_1_), PVA concentration (%, *w*/*v*) (X_2_), and organic/aqueous phase ratio (*v*/*v*) (X_3_). According to the ANOVA results, we determined the coefficients of the regression equations, the coefficients of determination, and the F-distribution (Equation (5), Figure 2, Table 2). The coefficient of determination (R^2^) and the F-test confirmed the adequacy of the mathematical model (Table 2). We applied the following polynomial equation (Equation (1)) to evaluate the effect of independent variables on NiTPP LC:

Y_1_ = 20.27 − 0.7X_1_ − 17.05X_2_ + 83.08X_3_ + 0.18X_1_X_2_ − 1.32X_1_X_3_ + 4.93X_2_X_3_ + 0.01X_1_^2^ + 3.56X_2_^2^ − 138.04X_3_^2^ (1)

According to the quadratic Equation (1), the O/W ratio was a positively significant factor: a positive linear relationship between NiTPP LC and the O/W ratio was confirmed as shown in the 2D and 3D plots (Figure 2). The reduction of the aqueous phase volume limited the organic solvent diffusion and led to the formation of NPs with an increased NiTPP LC. However, the PLGA and PVA concentrations were negative significant factors: the low PVA concentration facilitated the diffusion of the organic solvent and stabilized the NPs while the low PLGA concentration promoted rapid NP formation [48,49]. The PLGA-PVA and PVA-O/W pairs interacted positively, while O/W had a negative interaction with PLGA.

### 2.3. Particle Size

PS is another essential parameter affecting biological functions, such as NPs nonspecific entrapment with the reticuloendothelial system, renal clearance, and target cell accumulation. The size of the resulting particles has to be within a certain range in order to maximize the therapeutic efficacy. The PS of the formulated NiTPP-NPs was in a range from 272 to 700 nm (Table 1). The calculated coefficients of the regression equations and determination and F-distribution confirmed the adequacy of the mathematical model (Table 2). According to the ANOVA coefficients, we obtained a nonlinear quadratic equation (Equation (2)) for prediction of the response surface shape:

Y_2_ = 929.3 + 8.1X_1_ − 483.34X_2_ − 5133.22X_3_ + 1.42X_1_X_2_ − 44.4X_1_X_3_ + 1136X_2_X_3_ − 0.07X_1_^2^ + 111.64X_2_^2^ + 15,430.22X_3_^2^ (2)

We concluded that PS of NPs is proportional to X_1_, while factors X_2_ and X_3_ lack a direct correlation with PS (Figure 3). The PS increment along with an enlargement of the polymer loading during PLGA NPs preparation is a well-known fact [50]. However, the interaction of PVA with PLGA and the O/W ratio was positively significant. The reduction of the aqueous phase volume relative to the organic one led to a decrease in PS [51]. The described conditions probably stabilized the droplets of particles better in the aqueous phase and prevented their aggregation in the presence of a high PVA concentration [52,53]. As we expected, PLGA and the O/W ratio had a negative significant effect on PS.

### 2.4. Optimization

We calculated the optimal theoretical conditions for NP formulation based on the minimized PS and maximized NiTPP LC: the parameters included 5 mg of PLGA, 1.47% PVA, and 1:7 (organic/aqueous phase ratio). These conditions allowed us to prepare NPs with a predicted PS of 296.8 nm, NiTPP LC of 10.1%, and desirability of 0.835. We applied these parameters to prepare NiTPP-loaded NPs and analyzed them (Table 3). The comparison of the obtained and predicted data revealed the values’ proximity and low relative error. Relative error (RE) was calculated according to Equation (3):

RE = (A_prd_ − A_obs_)/A_prd_ (3)

where A_prd_ is the predicted data and A_obs_ is the observed data.

According to the results, the response surface model was valid and reproducible [54].

Further, we applied BBD analysis to optimize the formulation of MnClTPP- and CoTPP-loaded NPs as mentioned before. The best values of the dependent variables were provided with the same values of the independent variables (Table S3, Supplementary Materials). Perhaps the determination of such parameters is associated with some similar characteristics of MeP, for example, the solubility in solvent and the non-solvent volume. Rouhani and reported ZnTPP PLGA-NP optimization with the same value of factor C [54], as well as Lorenzoni and colleagues, who optimized Ga(III)-phthalocyanine PLGA-NPs [55]. These results may evidence the universality of certain factors for the formulation of NPs loaded with tetrapyrrole macroheterocyclic compounds.

We verified the surface response with the results obtained (Table 3). The data clearly showed that the Box–Benken method was suitable for the development of a method for MeP-loaded NP formulation.

### 2.5. Analysis of the MeP-NPs

Along with LC and PS, we considered EE, zeta potential, and PDI as the important parameters (Table 4). MnClTPP-loaded NPs possessed the maximum LC and EE and the minimum PS and PDI compared with other NPs. The NPs included NiTPP predominantly on the surface, while CoTPP and MnClTPP were entrapped mainly in the PLGA matrix. PS of the NPs allowed them to move freely via tumor vessels, avoiding absorption by macrophages and excretion by kidneys [56,57]. The CoTPP-NPs and NiTPP-NPs possessed a negative zeta potential due to PLGA terminal carboxyl groups and PVA. However, MnClTPP-loaded NPs revealed a positive surface charge that may be explained by MnClTPP’s presence on the NPs’ surface and a peculiar MnClTPP coordination center surrounded by an axial chloride ion. PDI was less than 0.200, indicating a tight NP size distribution.

### 2.6. Morphology

**Figure 4:**
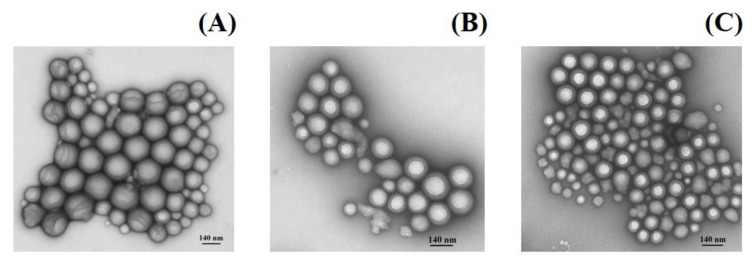
TEM microphotographs of MeP-loaded PLGA NPs: NiTPP (**A**), CoTPP (**B**), and MnClTPP (**C**).

The TEM micrographs of NPs displayed a smooth spherical shape with a narrow size distribution and dispersion without aggregation (Figure 4).

The structure of NPs corresponded to the core-shell type [58,59]. These results correlated with the dynamic light scattering data.

### 2.7. X-ray Diffraction (XRD) Study

**Figure 5:**
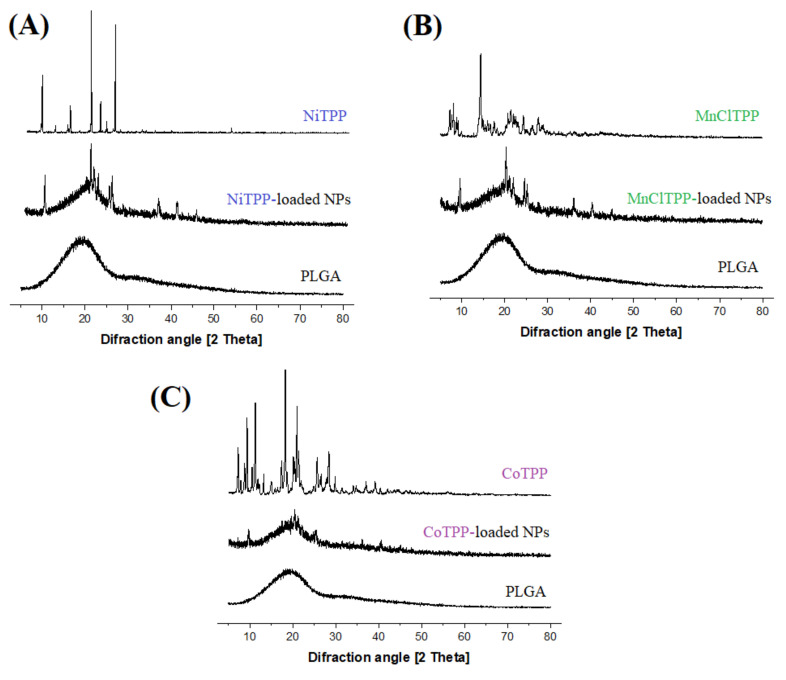
X-ray diffraction patterns of NiTPP (**A**), MnClTPP (**B**), and CoTPP (**C**). Each pattern (from top to bottom) displays the spectra of substance, MeP-loaded NPs, and PLGA.

Further, we analyzed the XRD patterns to study the physical state of pure MeP, PLGA, and NPs loaded with CoTPP, MnClTPP, and NiTPP. The sharp and intense peaks at 2-theta from 7° to 30° in the NiTPP and CoTPP XRD plots indicated their crystalline state (Figure 5A,C), which is in agreement with the results of Maclean et al. [60]. The peak at 2-theta of 14° evidenced the crystalline state of MnClTPP (Figure 5B). PLGA lacked intense peaks at 2-theta from 15° to 25°, displaying a dome-shaped region due to an amorphous state (Figure 5). These results agreed with those of previous studies [61]. The low relative amount of MeP loaded into NPs was expressed as a low intensity of XRD peaks, compared to free MeP. Probably, the PLGA XRD pattern partially masked the peaks of encapsulated MeP. Additionally, MePs in NPs could be of nano-range sizes and thus lack a long-range order, thus giving weaker lines in the XRD patterns. Nevertheless, MePs peaks were still recognizable. As a result, we concluded that the obtained samples were mostly in a crystalline rather than in an amorphous state.

### 2.8. Fourier Transform Infrared (FTIR) Spectroscopy

**Figure 6:**
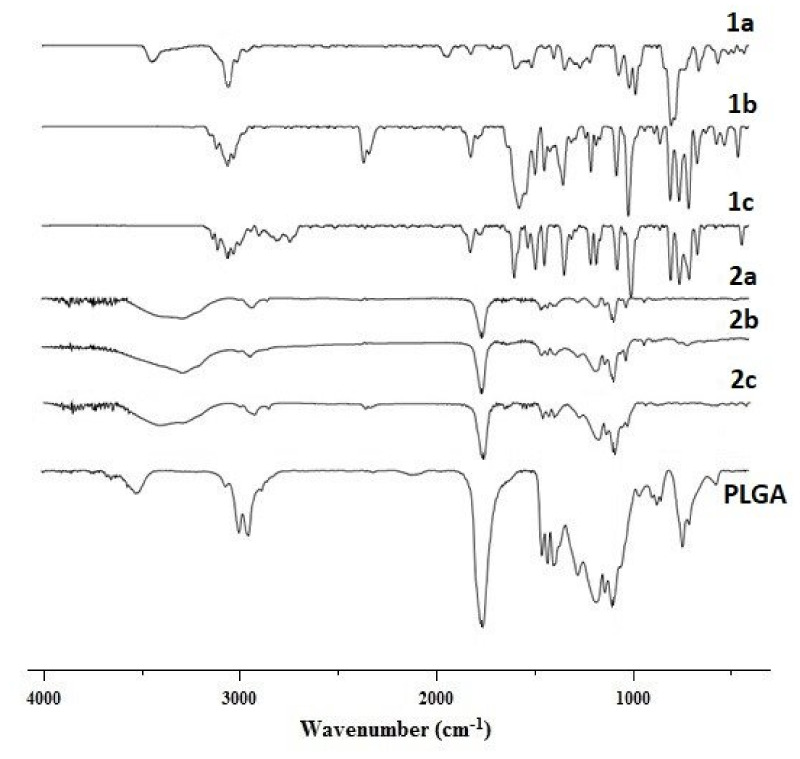
Comparative FTIR spectra of substances (NiTPP—1a, MnClTPP—1b, CoTPP—1c), NPs, containing MeP (NiTPP—2a, MnClTPP—2b, CoTPP—2c), and PLGA.

We applied FTIR spectroscopic analysis to reveal any chemical interactions between the substance and polymer (Figure 6). PLGA displayed characteristic peaks at 1758 cm^−1^ (C=O stretching), 1150 and 1300 cm^−1^ (C-C(=O)-O- stretching), 2950 and 3000 cm^−1^ (C-H stretching), and 3500 cm^−1^ (O-H stretching) [62].

MeP displayed peaks between 2900 and 3100 cm^−1^ corresponding to stretching vibrations =C-H. MnClTPP and CoTPP displayed peaks at 999–1012 cm^−1^, and NiTPP displayed a peak at 1007 cm^−1^ attributed to planar deformations in porphyrins [63,64,65]. Previously, several tetraphenylporphyrin derivatives with divalent metals displayed similar peaks [64].

The spectra of MeP-loaded NPs were similar to PLGA, except the shift of the peak characterizing O-H stretching vibrations from 3500 to 3288–3399 cm^−1^.

We observed no significant shifts for other bands suggesting no other specific chemical interactions between a drug and the polymer while encapsulating.

### 2.9. Interactions between PLGA and MeP

**Figure 7:**
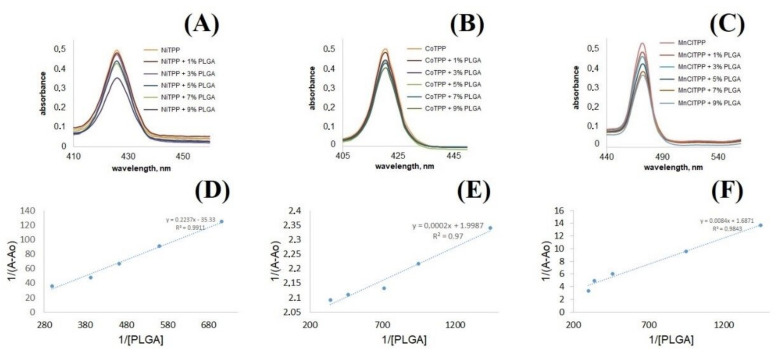
UV-vis spectra of NiTPP (**A**), CoTPP (**B**), and MnClTPP (**C**). Scatter plots with trend lines plotted in the coordinates Y = 1/[PLGA] and X = 1/(A − A_o_) for PLGA complexes with NiTPP (**D**), CoTPP (**E**), and MnClTPP (**F**).

We analyzed the binding constants to evaluate the affinity of MnClTPP, NiTPP, and CoTPP to PLGA. According to Equation (4), PLGA was diluted in 3 mL of chloroform, methylene chloride or acetone (the solvent used during NP formulation) to achieve a 1, 3, 5, 7, and 9% mass concentration. The binding constants of the complexes were assessed according to MeP absorbance reduction (MnClTPP at 473 nm, CoTPP at 430 nm, NiTPP at 434 nm) (Figure 7A–C):

(4) $$W=\frac{m\;\left(\mathrm{PLGA}\right)\;}{m\left(\mathrm{PLGA}\right)+\;m\left({\mathrm{CHCl}}_{3}\;\mathrm{or}\;{\mathrm{CH}}_{2}{\mathrm{Cl}}_{2}\;\mathrm{or}\;C_{3}H_{6}O\right)+\;m\left(\mathrm{MeP}\right)}$$

The masses of PLGA samples were recalculated (Equation (5)) by applying corresponding mass concentrations according to Equation (4), considering the mass of MeP dissolved in chloroform, methylene chloride, or acetone:

(5) $$m\;(\mathrm{PLGA})=\frac{W\;\times \left(m\left(\mathrm{MeP}\right)+\;m\left(S\right)\right)\;}{1-\;W}$$

where W is the mass concentration (%), m(MeP) is the MeP mass (g), and m(S) is the organic solvent (acetone, chloroform, methylene chloride) mass (g).

The molar concentration of the polymer ([PLGA], M/L) was calculated according to Equation (6) (PLGA molar mass was 17,000–21,000 Da [66]):

(6) $$[\mathrm{PLGA}]=\frac{m\left(\mathrm{PLGA}\right)}{\mathrm{Mw}\;\times \;\mathrm{Vmix}}$$

where m(PLGA) is the PLGA mass (g), M_W_ is the molar mass concentration of PLGA (Da), and V_mix_ is the total volume of suspension containing MeP and PLGA (L).

The absorption spectra of the MeP and PLGA mixture (Figure 7A–C) clearly demonstrated a decrease in the intensity of CoTPP, NiTPP, and MnClTPP caused by PLGA in the range 220–600 nm.

The binding constant was calculated according to the Benesi–Hildebrand Equation (7) by applying the resulting PLGA molar concentration and MeP optical density [67]:

(7) $$1/(A-A_{o})=\frac{1}{\mathrm{Ka}\left(\mathrm{Amax}-\mathrm{Ao}\right)\;\left[\mathrm{PLGA}\right]\;}+\frac{1}{\mathrm{Amax}-\mathrm{Ao}}$$

where A_o_ is the MeP absorption in the absence of PLGA, A is the MeP absorption in PLGA’s presence, and A_max_ is the MeP absorption in the presence of 9% PLGA. *K_d_* represents tgα after plotting the linear dependences of 1/(A − A_o_) and 1/[PLGA] (Figure 7D–F) (Table 5).

**Figure 8:**
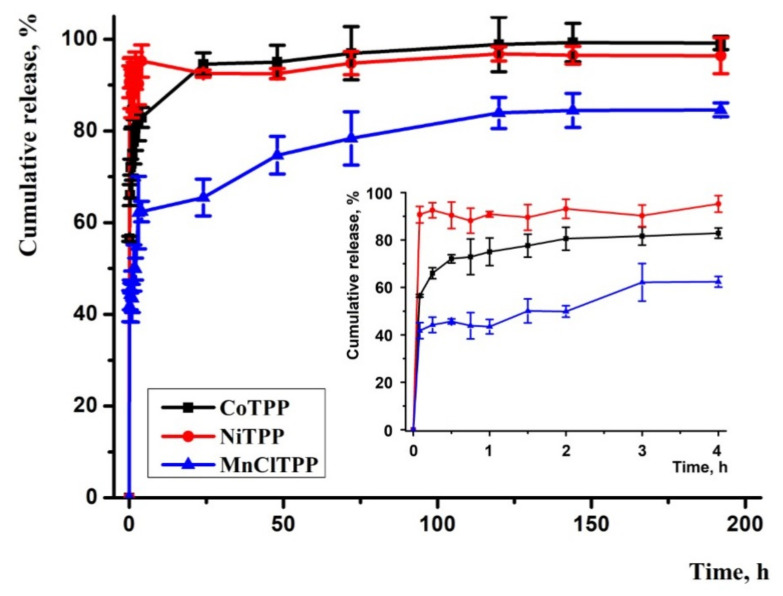
In vitro release study of MeP-loaded NPs in phosphate buffer saline pH 7.4 with 0.1% Tween 80. Each point shows the mean ± S.D. (*n* = 3).

The data confirmed strong binding of MeP with PLGA and the formation of PLGA-MeP complex. The binding constants correlated with EE and LC data. Moreover, the complex formation could affect the MeP release from the polymer matrix (Figure 8) [68]. We assumed that a lower binding constant of PLGA with MeP determined a longer release. These calculations allowed us to predict EE in PLGA NPs and the release behavior of MeP.

### 2.10. Hemolytic Activity Study

The nanocarrier hemolysis assay has become an important procedure for evaluation of the biocompatibility of intravenously administered substances [69,70]. Some reports described NPs’ hemolytic activity due to damage of the erythrocyte membrane following severe side effects (anemia, kidney injury) caused by hemoglobin release [71].

In our study, blank NPs and CoTPP-loaded NPs lacked hemolytic activity after incubation with blood samples for 3 h (Table 6). However, NiTPP-loaded NPs demonstrated a negligible toxic effect (hemolytic rate was 6.5%) at the maximal concentration [72]. The partially surface-trapped NiTPP could explain this effect. Several reports described NiTPP’s ability to adhere and disrupt the membrane of red blood cell and induce hemolysis [73,74]. The MnClTPP-loaded NPs induced strong hemolysis at high concentrations of 0.09–25 mg/mL. The hemolytic activity of Mn-loaded NPs could be explained by their positive surface charge [75,76]. However, MnClTPP-NPs lacked hemolytic activity at concentrations lower than 0.09 mg/mL, demonstrating biocompatibility and potential for application in nanomedicine [77,78,79,80,81]. Thus, MeP-loaded NPs demonstrated good potential for application in drug delivery systems as blood-compatible vehicles.

### 2.11. In Vitro Drug Release

We analyzed the release according to the method of Abouelmag et al. using a phosphate-buffered saline (PBS) pH 7.4 containing 0.1% Tween 80 (Figure 8) as a release medium [82]. Tween 80 as emulsifier proved useful to maintain the hydrophobic-released drug’s stability in the release media [83].

The results indicated that MnClTPP-NPs exhibited a biphasic release profile similar to that previously reported by Xu and colleagues [84]. A fast MnClTPP release step continued during the first 15 min followed by gradual release that was associated with the partial MeP presence on the NPs’ surface. After 25 h, a MeP spike release repeated and reached a plateau. The profiles of NiTPP and CoTPP release were also biphasic: a rapid phase continued during the first 30 min and switched to a prolonged release of the substance. The release rates of CoTPP-NPs and NiTPP-NPs almost coincided, which is explained by similar PSs and LC. These results agreed with the binding constant data. A high binding constant of MnClTPP-PLGA complex contributed to a longer MnClTPP release in contrast with NiTPP and CoTPP-loaded NPs, characterized by low binding constants. Notably, the sharp release of NiTPP could be explained by low EE. Sharp release profiles of MeP will contribute to the rapid MeP release in the intracellular space and time reduction for AA addition to achieve an antitumor effect. Overall, the MeP release profiles were consistent with those in previous reports and correlated with the binding constants, EE, and LC [85,86,87,88,89].

We determined the MeP release mechanisms by fitting the data obtained by applying several release kinetic models (Table 7). It was found that the Korsmeyer-Peppas model better described the kinetics of MeP release and demonstrated the highest correlation coefficients (R^2^) [90]. According to the Korsmeyer–Peppas model, the release rate constants were: *k_к_* = 91.3 h^−1^ for NiTPP, *k_к_* = 74.3 h^−1^ for CoTPP, *k_к_* = 49.4 h^−1^ for MnClTPP [91]. The release exponent values were *n* = 0.01 for NiTPP, *n* = 0.06 for CoTPP, and *n* = 0.11 for MnClTPP, and less than 0.43, indicating Fickian diffusion of MeP [92]. These results corresponded to previously reported data describing lipophilic substance release mechanisms [93,94,95,96].

### 2.12. In Vitro Cytotoxicity of MeP and MeP-NPs

**Figure 9:**
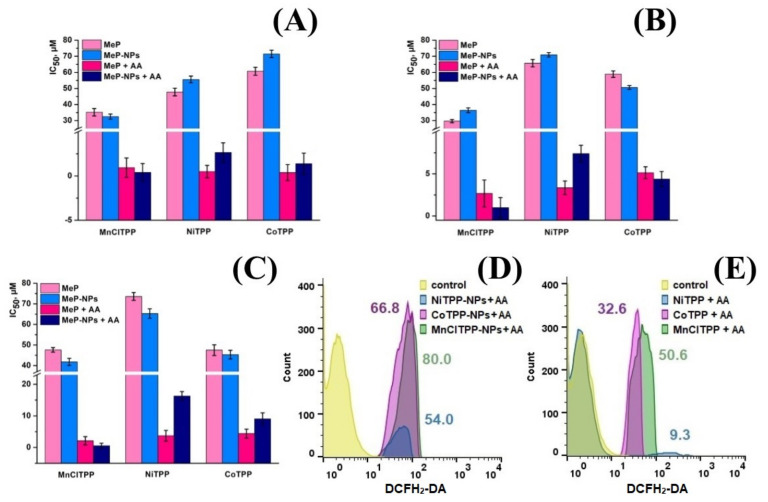
Cytotoxicity of MePs and MeP-NPs against HeLa cells (**A**), MCF-7 cells (**B**), and SK-OV-3 cells (**C**) analyzed by MTT assay after 72 h of incubation. The percentage of cell viability was determined relative to a viable control. Flow cytometry analysis of MCF-7 cells treated with the combination of AA and MeP-NPs (**D**) and the combination of AA and MeP (**E**).

Figure 9 shows the IC50 values of MeP-NPs and AA combination against HeLa, MCF-7, and SK-OV-3 cells. We selected the AA concentration according to the results of our previous study [97]. The MeP combination with AA exhibited significant cytotoxic activity against all examined cell lines; AA, at the same concentrations, lacked cell growth inhibition (Figure S5). However, MeP and MeP-NPs displayed weak cytotoxicity, evidencing cell death as a result of catalytic reaction between MeP and AA. Figure 9A,B displays that MnClTPP-NPs/AA increased the cytotoxic effect compared to MnClTPP/AA. Notably, the MnClTPP/AA and MnClTPP-NPs/AA combinations were significantly effective in comparison with other MePs.

The cytotoxic activity of NiTPP and CoTPP was similar against all cancer cell lines. Interestingly, CoTPP/AA and NiTPP/AA revealed toxicity similar to that of the nanoformulation-based combinations. We speculated that NiTPP’s and CoTPP’s rapid release from NPs does not allow us to detect a difference between MeP and MeP-NPs. The results obtained may indicate that NiTPP and CoTPP possess similar catalytic characteristics and exhibit lower antitumor activity than MnClTPP.

Noteworthy, the LC, PS, and MeP release profiles affect the cytotoxic activity of NPs [49]. A biphasic release profile of MnClTPP, small PS, and high LC increase NPs’ cytotoxicity and accumulation selectivity, while rapid CoTPP and NiTPP release as well as low LC led to minor differences in antitumor activity [98]. Nevertheless, PS of formulated NPs allowed free circulation in the bloodstream, LC was sufficient to achieve a toxic dose, and the fast MeP release in combination with AA rapidly induced ROS formation. Overall, these results suggested that AA combinations with the selected MeP are promising for cancer treatment.

### 2.13. ROS Formation Analysis

The antitumor effect of the catalyst system was achieved via ROS formation during the redox reaction between MeP and AA. Therefore, we analyzed the intracellular ROS level during treatment with the catalyst system using DCFH_2_-DA (2,7-dichlorofluorescein diacetate) (Figure 9D,E). We observed an enhancement of green fluorescence after MnClTPP/AA and MnClTPP-NPs/AA treatment, suggesting ROS formation during the catalyst reaction. The NiTPP and CoTPP activity was similar, but the CoTPP/AA and CoTPP-NPs/AA combinations more efficiently induced ROS formation than NiTPP/AA and NiTPP-NPs/AA. These results agreed with those of previous studies of Mn- and Co- porphyrins [28,99]. Nevertheless, the results of flow cytometry analysis demonstrated that MeP encapsulation in a polymer matrix promoted their intracellular accumulation and ROS generation, leading to cell death.

### 2.14. Acute Toxicity Analysis

Acute toxicity analysis revealed MeP-NPs’ safety up to a dose of 200 mg/kg, whereas the MePs demonstrated higher toxicity (Table 8). Moreover, dotted lung hemorrhages were observed in the mice that had received 25 and 50 mg/kg of MePs.

### 2.15. In Vivo Pharmacokinetic Study

**Figure 10:**
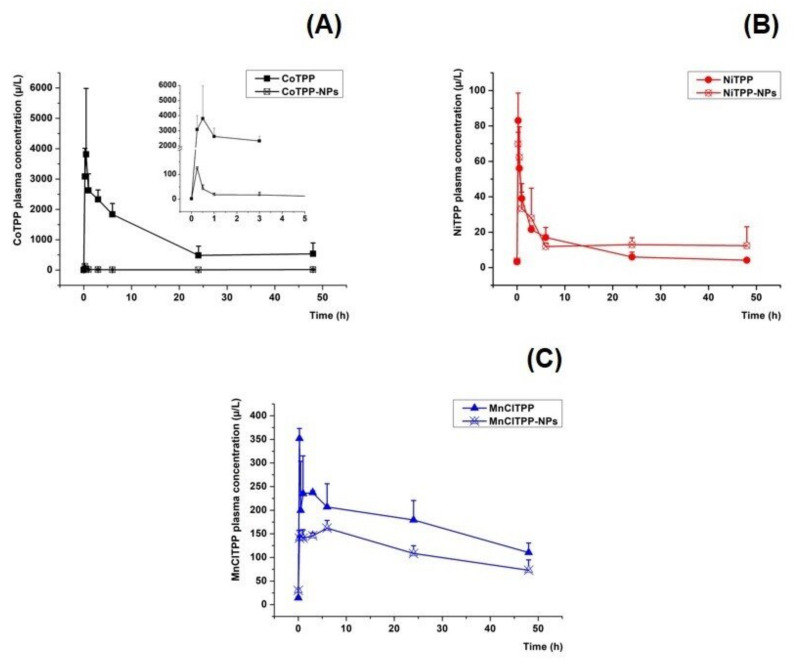
Pharmacokinetic plasma concentration–time curves of MeP and MeP-loaded NPs: (**A**) CoTPP formulations at a CoTPP dose equivalent to 12.5 mg/kg, (**B**) MnClTPP formulations at a MnClTPP dose equivalent to 6.3 mg/kg, and (**C**) NiTPP formulations at an NiTPP dose equivalent to 12.5 mg/kg (mean ± S.D., *n* = 3).

The MeP and MeP-NPs were administered via the lateral tail vein at tahe dose of 12.5 (NiTPP, CoTPP, and corresponding NPs at the equivalent dose) and 6.3 mg/kg (MnClTPP and MnClTPP-NPs at the equivalent dose). The animals lacked toxic effects after injection and during the experiment. We chose the lower MnClTPP dose according to previously reported results of an acute toxicity study [97]. The MeP and MeP-NPs profiles exhibited a biphasic behavior (Figure 10). MnClTPP-NPs and CoTPP-NPs displayed a lower MeP plasma concentration after injection compared to the free substances during the distribution and elimination phases. However, the plasma concentrations of NiTPP-NPs and NiTPP displayed almost identical profiles. The pharmacokinetic parameters of NiTPP (12.5 mg/kg), CoTPP (12.5 mg/kg), MnClTPP (6.3 mg/kg), and NPs at the equivalent doses are represented in Table 9.

Surprisingly, MnClTPP-NPs displayed decreased AUCinf, t1/2, and MRT compared to free MnClTPP, which may indicate rapid tissue distributionof MnClTPP-NPs. Moreover, due to slow MnClTPP release and the high binding constant, the concentration peak of MnClTPP could coincide with a burst release and lead to a prolonged long-term MnClTPP release (Section 2.8) in tissues, since only 80% of MnClTPP was released after 180 h. Probably, the high association of free MnClTPP with serum albumin was responsible for the long blood circulation [100].

In the case of CoTPP-NPs, we observed an increased Vd, while AUCinf values were significantly lower compared to the control substance. This pattern also indicated a rapid distribution of CoTPP-NPs in tissues.

The encapsulation weakly affected the pharmacokinetic parameters of the NiTPP-NPs, which agreed with the release kinetics data and low binding constant (Section 2.8). Additionally, the main fraction of the encapsulated NiTPP was surface entrapped, providing rapid release and subsequent association with blood proteins.

The encapsulation of MnClTPP and CoTPP affected the pharmacokinetic profile of NPs; rapid elimination from the bloodstream of MnClTPP-NPs and CoTPP-NPs, compared to free compounds, evidenced more specific tissue distribution due to the shorter circulation time of lipophilic compounds. This behavior could benefit the MeP safety profile and efficacy, as we reported previously [46,101]. Further toxicological studies and determination of the formulated NPs’ safety profile and biodistribution will be able to provide a full justification of the plasma pharmacokinetics profiles.

MeP are widely used in medicine as photosensitizers, sonodynamic agents, and antitumor drugs; therefore, the development of effective and safe MeP formulations is an urgent task for researchers [17,18,21]. PLGA NPs are biocompatible and reduce toxic side effects of drugs via selective tumor accumulation and enhanced bioavailability, thus we selected PLGA nanocarriers for MeP encapsulation [36]. Specific attention should be paid to PS and LC of MeP when designing NPs [53,54]. NPs less than 800 nm accumulate in tumor tissues [33]. The level of LC affects the dose regime and the amount of drug administered [54]. Thus, selective accumulation in tumor tissues and reduced drug dose are crucial parameters for reducing toxic effects. Therefore, when optimizing the NPs formulation method, we focused on the low particle size and high LC of MeP. In this study, we applied the BBD, since this method is widely used to optimize the NPs formulation processes and demonstrated high prediction accuracy for NPs characteristics [45,91]. To obtain MeP-loaded NPs, we used nanoprecipitation and single emulsion-solvent evaporation methods, since they are commonly used for MeP encapsulation into a polymer matrix [102,103,104]. During the optimization process, we found that each MeP was characterized by a different influence of factors (PLGA mass, PVA concentration, O/W phase ratio) on PS and LC. However, the variation range of these factors was quite narrow, which can be explained by the similar structure of the investigated MeP. We suggest that this optimized preparation method can be suitable for other tetraphenylporphyrins.

We found that the MeP release pattern from PLGA NPs was biphasic. MeP-loaded NPs were characterized by a sharp burst release phase during the first 30 min. Similar results were described for PLGA nanoparticles containing lipophilic drugs [105]. It was shown that MeP encapsulation demonstrated an absence of hemolytic activity and potential for application in nanomedicine. The most common problem using free MeP is acute toxicity [106]. The present study revealed high antitumor activity of **MeP-NPs** in combination with AA compared to free MePs. In addition, we showed that MnClTPP and CoTPP-loaded NPs efficiently induced tumor cell death, which is consistent with previous results obtained by other authors who studied Co- and Mn-porphyrins activity in combination with AA [28,98]. Taken together, the BBD method contributed to the development of MeP-loaded NPs with the desired characteristics, and MeP encapsulation improved their safety, compatibility, and preserved antitumor activity.

## 3. Materials and Methods

### 3.1. Materials

Poly(d,l-lactide-*co*-glycolide) (PLGA polymer with carboxylic terminal group, 50/50 of inherent viscosity midpoint 0.4 dL/g; MW 17,000–21,000), was purchased from LACTEL Absorbance Polymers (Birmingham, AL, USA). D-mannitol, Polyvinyl alcohol (PVA, MW 30,000–70,000), Sodium dodecyl sulfate (SDS), Triton X-100, and N-butyl alcohol were purchased from Sigma-Aldrich (St. Louis, MO, USA). Chloroform, methylene chloride, and acetone were purchased from Ruskhim (Moscow, Russia). Deuterochloroform (CDCl_3_) and Dideuteromethylenechloride (CD_2_Cl_2_) were purchased from Cambridge Isotope, Canada, UDA. Dimethyl sulfoxide (DMSO) and phosphate-buffered saline (PBS) were purchased from Amreso (Solon, OH, USA). Nitric acid was purchased from Chimmed (Moscow, Russia) and Tween 80 was purchased from Serva (Heidelberg, Germany).

### 3.2. Synthesis of MeP

Tetraphenylporphyrins were synthesized using the condensation reaction between benzaldehyde and pyrrole according to the published procedure (Figure 1A) [107]. The coordination of metal ions was carried out by the method reported previously [108]. The yield of the NiTPP was 58%, ^1^H NMR: (400 mHz, CDCl_3_) δ ppm: 7.64 (12H, p-phenyl), 7.66 (8H, m-phenyl), 7.97 (8H, o-phenyl), 8.75 (s, 8H, Hβ-pyrrolic). MnClTPP was yielded with 69%, ^1^H NMR: (400 mHz, CD_2_Cl_2_) δ ppm: 7.33 (12H, p-phenyl), 8.32 (8H, m-phenyl), not available (o-phenyl), −21.95 (s, 8H, Hβ-pyrrolic). For CoTPP, the yield was 64%, ^1^H NMR: (400 mHz, CDCl_3_) δ ppm: 7.97 (4H, p-phenyl), 8.16 (8H, m-phenyl), 13.11 (8H, o-phenyl), 16.45 (s, 8H, Hβ-pyrrolic).

### 3.3. Preparation of Nanoparticles

MnClTPP-NPs and NiTPP-NPs were formulated via the single emulsion-solvent evaporation method (Figure 1C) [50]. This method is suitable for encapsulation of water-insoluble drugs [50]. Briefly, 10 mg of MnClTPP or NiTPP and mass of PLGA were dissolved in 2.5 mL of chloroform (for MnClTPP) and methylene chloride (for NiTPP), as the organic phase. Then, the mixture was added drop-by-drop to PVA solution. The emulsion was sonicated (Labsonic U.B.Braun, Melsungen, Germany) and then evaporated during 50 min under reduced pressure to remove the organic solvent (IKA HB10, Staufen im Breisgau, Germany). Further, NPs were collected via centrifugation for 1 h at 15,000× *g*, 4 °C and washed twice with distilled water following centrifugation at 17,000× *g* for 30 min (BECKMAN J2-2, Palo Alto, CA, USA). After centrifugation, the sediment was resuspended in 10 mL of distilled water; filtered using a glass filter with a porosity of 47–111 μm, and 10% (*w*/*w*) of D-mannitol was added to the suspension. The resulting NPs were lyophilized and stored at 4 °C until further use.

CoTPP nanoparticles were synthesized via the nanoprecipitation method (Figure 1D) [88,102]. The aforementioned amounts of CoTPP and PLGA were dissolved in 2.5 mL of acetone. The CoTPP-polymer mixture was added dropwise to PVA solution and kept under stirring for 20 min. Further, the solvent was evaporated, the NPs washed from PVA, centrifuged, and lyophilized as described earlier for MnClTPP-NPs and NiTPP-NPs.

### 3.4. Box–Behnken Experimental Design

We applied a Box–Behnken experimental design (BBD) with response surface methodology for statistical optimization of MeP-loaded NP preparation [45]. The design of the experiment was a 2-level 3-factor design with five repetitions of the central factors, which required 17 experiments to obtain particles containing MeP [91]. We chose the amount of PLGA (***X*_1_**), concentration of the emulsion stabilizer (PVA) (***X*_2_**), and organic to aqueous phase ratio (O/W) (***X*_3_**) as independent factors influencing the physicochemical characteristics of the particles, as noted earlier.

MeP LC into PLGA (Y_1_) and particle size (PS) (Y_2_) were selected as dependent variables, which are the main factors influencing the dosage regimen of MeP and the effectiveness of intratumoral accumulation [33,34]. The optimization was carried out at two levels (low and high) as mentioned in Table 10. The interaction of the independent variables and responses was evaluated using the following regression equation (Equation (8)) [109]:

Y = β_0_ + β_1_X_1_ + β_2_X_2_ + β_3_X_3_ + β_1,2_X_1_X_2_ + β_1,3_X_1_X_3_ + β_2,3_X_2_X_3_ + β_11_ X_1_^2^ + β_22_ X_2_^2^ + β_33_ X_3_^2^ (8)

where Y is the dependent variable; β_0_ is the mean response value of 17 runs; β_1_, β_2_, β_3_ are the regression coefficients for each factor; β_1,2_, β_2,3_, β_1,3_ are the interaction coefficients of the equation; and β_1,1_, β_2,2_, β_3,3_ are the significance of the coefficients.

The determination of the independent factors with the greatest impact on the response functions, the interaction level between factors, and statistical values was performed by statistical analysis using Design-Expert software (Stat-Ease Inc., version 7.0; Design-Expert software; Minneapolis, MN, USA).

After determining the factors and interactions affecting the response surface, NP optimization was performed using numerical analysis. The criteria for the optimized technique were the maximum MeP LC and minimum PS [45]. Among the results proposed by the program solutions, the value that was closer to the optimal (since the probability of achieving the desired result is higher) was selected. We applied the calculated values of independent variables to formulate optimized particles and analyzed MeP LC and PS. The observed results were compared with the predicted data and the percentage of relative error was calculated.

### 3.5. Characterization of NPs

#### 3.5.1. Encapsulation Efficiency (EE) and MeP Loading Content (LC)

EE and LC calculation is a critical stage in NP analysis, evaluating the MeP amount in NPs’ core [54]. The EE of MeP was determined by dissolving 4 mg of NPs in 4 mL of distilled water followed by centrifugation at 5000× *g* for 3 min and subsequent lyophilization. The optical density of the sediment and supernatant was assessed at 465 nm for MnClTPP, 433 nm for NiTPP, and 428 nm for CoTPP. The entrapment efficiency of MeP (EE) was calculated according to Equation (9) [45]:

(9) $$\mathrm{EE}\;(\%)=\frac{\mathrm{The}\;\mathrm{mass}\;\mathrm{of}\;\mathrm{MeP}\;\mathrm{in}\;\mathrm{the}\;\mathrm{nanoparticles},\;\mathrm{mg}}{\mathrm{The}\;\mathrm{mass}\;\mathrm{of}\;\mathrm{the}\;\mathrm{nanoparticles}\;\mathrm{weight},\;\mathrm{mg}\;}*100\%$$

MeP LC corresponded to the optical density of 4 mg of lyophilized NPs in 4 mL of DMSO. Subsequent absorption analysis was performed as indicated earlier and LC calculated according to the following Equation (10) [97]:

(10) $$\mathrm{LC}\;(\%)\;=\;\frac{\mathrm{Absorbance}*V_{\mathrm{DMSO}}}{\varepsilon *m_{\mathrm{sample}}}*100\%$$

where V_DMSO_ is the total volume of suspension containing NPs (L), ε is the extinction coefficient of MeP in DMSO (ε = 116,280 M^−1^ cm^−1^ for MnClTPP, ε = 36,797 M^−1^ cm^−1^ for NiTPP, ε = 87,448 M^−1^ cm^−1^ for CoTPP), and m_sample_ is the NPs mass (g).

#### 3.5.2. Size (PS), Zeta Potential, and Polydispersity Index (PDI) Measurement

The size and PDI of MeP-NPs were analyzed by dynamic light scattering (DLS) and the zeta potential was measured using electrophoretic light scattering (Nano-ZS ZEN 3600, Malvern-Instruments, Worcestershire, UK) [48]. The samples were diluted with distilled water to reach a final concentration of 1 mg/mL and three independent samples were measured [105].

#### 3.5.3. Nanoparticle Morphology

The morphology of MeP-NPs was analyzed using transmission electron microscopy (TEM) (Tecnai Osiris, FEI, Hillsboro, OR, USA) [58]. A drop of NP water suspension (1 mg/mL) was placed on a 3 mm copper grid covered with formvar film and dried for 30 min.

#### 3.5.4. X-ray Diffraction Study (XRD)

The crystallinity of MeP, PLGA, and MeP-NPs was determined by the diffraction pattern on an X-ray diffractometer (Philips PW1710 (Eindhoven, The Netherlands), operating with Cu Kα1 radiation, λ = 1.5406 A°, 50 kV voltage, and current 250 mA. The diffraction pattern was determined in the area 5° < 2-theta < 80°, using a stepwise method (0.2°/s) [61].

#### 3.5.5. Fourier Transform Infrared Spectroscopy (FTIR)

FTIR spectra were recorded for MeP, PLGA, and MeP-NPs using an FTIR spectrometer to confirm MeP loading into NPs (FTIR, Bruker Equinox 55, Bruker, Billerica, MA, USA) [86]. NPs were lyophilized prior to FTIR spectroscopic analysis [88]. The samples were prepared using micronized KBr (FTIR-grade, Merck, Germany), heated up to 100 °C for 2 h prior to pellet preparation. Then, 1% of a sample (relative to the KBr amount) was mixed and pressed with KBr powder into a conventional 13 mm diameter disk (thickness 1.5 mm) under 5000 Psi of pressure for 2 min; FTIR spectra were scanned from 400 to 4000 cm^−1^.

### 3.6. In Vitro Release Study

The time-dependent release of MeP was analyzed by dissolving 3 mg of NPs in 1 mL of PBS (0.01 M, pH 7.4) and incubation in a dark glass bottle containing 40 mL of PBS (0.01 M, pH 7.4) and 0.1% (*w*/*v*) Tween 80 solution. The probes were kept on a shaker incubator at 90 rpm and 37 °C. Aliquots of 1 mL of media were taken at 0, 0.25, 0.5, 0.75, 1, 1.5, 2, 3, 4, 24, 48, 72, 120, 144, and 192 h and replenished with 1 mL of fresh PBS. The collected aliquots were centrifuged for 3 min at 5000× *g* at 37 °C. The supernatants were removed and lyophilized as well as the sediments [82,83]. The amount of MeP released at each time point was calculated as described above according to optical absorption.

### 3.7. Interactions between PLGA and MeP

The analysis of MeP binding with PLGA was performed spectrophotometrically. PLGA 1, 3, 5, 7, and 9% *m*/*w* solutions in 3 mL of chloroform, acetone, or methylene chloride were prepared. Then, 30 μg of MeP dissolved in 30 μL of chloroform (MnClTPP), acetone (CoTPP) or methylene chloride (NiTPP) were added dropwise to a corresponding polymer solution. After 10 min, the absorption analysis of PLGA-MeP complexes was performed using a Spectronic Heλios α spectrophotometer (Thermo Fisher Scientific, Waltham, MA, USA) in the range of 200–600 nm against chloroform, acetone, or methylene chloride [83]. The optical density of MeP-PLGA complex was determined at 473 nm (MnClTPP), 434 nm (NiTPP), and 430 nm (CoTPP).

### 3.8. Hemolytic Activity Study

During the hemolysis assay, detection of oxidized forms of hemoglobin (hemichromic method) was performed. The blood was centrifuged at 358× *g*, 4 °C for 5 min. The supernatant was aspirated and replaced with 0.001 M PBS. This step was repeated three times. The following 0.001 M PBS addition led to a 1/5 dilution of red blood cells. Blood samples were incubated with blank NPs, MnClTPP-NPs, CoTPP-NPs, and NiTPP-NPs at concentrations of 25, 2.5, and 0.25 mg/mL for 3 h at 37 °C using an orbital shaker (with a blood:sample ratio 1:1). Because of MnClTPP-NPs’ pronounced hemolytic activity, we tested the additional concentrations of 0.09 mg/mL and 0.009 mg/mL. The positive control sample contained 1% Triton X-100 (100% hemolysis), while 0.001 M PBS was used as a negative control [70].

After incubation, the intact erythrocytes were separated by centrifugation at 2240× *g*, 4 °C for 5 min. The supernatants were transferred into a flat-bottom 96-well plate; SDS was added to each sample to the final concentration of 0.06%, inducing the formation of hemichromes. The hemolysis was calculated by comparing the samples’ absorbance at 540 nm with positive and negative controls (Equation (11)) [72]:

(11) $$\mathrm{Hemolysis}\;(\%)\;=\;\frac{Asamp\;-\;Aneg}{Asamp\;-\;Apos}*100\%$$

where *A_samp_* is the optical density of sample (mean value), *A_neg_* is the optical density of the negative control (mean value), and *A_pos_* is the optical density of the positive control (mean value).

### 3.9. Cell Culture

The cancer cell lines HeLa (human cervical carcinoma), MCF-7 (human breast adenocarcinoma), and SK-OV-3 (human ovarian carcinoma) were purchased from the American Type Culture Collection (ATCC, Manassas, VA, USA). Cells were maintained in DMEM medium supplemented with 10% fetal bovine serum and gentamycin (50 μg/mL). Cells were maintained in plastic 25 cm^2^ cell culture flasks at 37 °C in a humidified atmosphere containing 5% CO_2_. HeLa, MCF-7, and SK-OV-3 cells were plated before reaching 80% confluence using trypsin/EDTA solution.

### 3.10. Cytotoxicity Assay on MCF-7, SK-OV-3, and HeLa Cells

MCF-7, SK-OV-3, and HeLa cells were plated in 96-well plates (5000 cells per well) one day before the experiment and incubated under standard conditions. MeP and MeP-NPs were added in triplets in the concentration range 0.125–100 μM (according to the MeP concentration) and incubated for 2 h. To determine cytotoxicity, cells were treated with AA alone or in combination with MeP in a molar ratio of 1:20 to MeP and then incubated for 72 h [83]. Cell survival was determined using the standard MTT assay [110]. Cell viability was determined as the percent of untreated control. Mean survival values were calculated in Excel (Microsoft Corporation, Redmond, Washington, DC, USA)) and data visualization was performed in OriginPro (version 2020b, OriginLab Corp., Northampton, MA, USA).

### 3.11. Intracellular ROS Measurement

Qualitative ROS generation analysis was performed using a flow cytometer. Briefly, MCF-7 cells at a density of 1 × 10^5^ (1 mL/well) were seeded and incubated with 30 μM MeP for 2 h at 37 °C. After 2 h of AA treatment followed by 20 min 20 µM DCFH_2_-DA staining, cells were rinsed twice with PBS, collected in a 1 mL centrifuge tube, and immediately measured for fluorescence intensity (λex 488 nm, λem 525 for DCFH_2_-DA) [111].

### 3.12. Acute Toxicity Analysis

All experimental procedures with animals (mice and rats) were performed according to the University Ethics Committee for the use of experimental animals and animal studies were carried out as per the guidelines of the European Medicines Agency, Amsterdam, the Netherlands. Female Balb/c mice (body weight 20–22 g) were supplied by Stolbovaya animal facility (Branch of the Scientific Center of Biomedical Technologies of the Federal Medical Biological Agency (Moscow, Russia)). The animals were kept under controlled conditions (temperature of 22 ± 2 °C and relative humidity of 50 ± 10%) with free access to water and standard pellet feed.

MeP and MeP-NP acute toxicity analysis was accomplished using randomly divided mice groups (*n* = 3). The samples were diluted in PBS and injected (200 μL) via the lateral tail vein. Due to poor water solubility, MePs were preliminarily dissolved in DMSO and Tween 80: the final solution represented 5% *v*/*v* DMSO and 2% Tween 80 *v*/*v* in PBS. Survival was registered for 24 h after injection [83].

### 3.13. In Vivo Pharmacokinetic Study

Female Wistar rats (body weight 220–240 g) were supplied by Stolbovaya animal facility (Branch of the Scientific Center of Biomedical Technologies of the Federal Medical Biological Agency (Moscow, Russia)). The animals were kept under controlled conditions (temperature of 22 ± 2 °C and relative humidity of 50 ± 10%) with free access to water and standard pellet feed.

The MeP and MeP-NPs pharmacokinetics study was accomplished using randomly divided rat groups (*n* = 3). Blood samples (50 μL) were collected before injection and after at different time points: 0.25, 0.50, 1.0, 3.0, 6.0, 24.0, and 48.0 h. The blood samples were diluted to 1.0 mL and analyzed with ICP-MS (Agilent 7500C, Agilent Technologies, Tokyo, Japan) [112]. The sample introduction system consisted of a robust Babington nebulizer with a Scott spray chamber (Agilent Technologies, Tokyo, Japan) cooled by a Peltier element (2 °C). The data were acquired and processed with the ICP-MS ChemStation (Agilent Technologies, version G1834B; Santa Clara, CA, USA) software package. The internal standard method was applied in order to take into account the effect of the matrix; Rh was used as internal standard. For dilution of the whole blood samples and standard solution, the mixture of 0.1% Triton X-100–1% HNO_3_ was used. The same solution was used as the mobile phase of the mass spectrometer.

The pharmacokinetic parameters of MeP were estimated using standard non-compartmental methods [113]. The volume of distribution (Vd), mean residence time (MRT), elimination half-life (t_1/2_), and area under the plasma concentration–time curve from 0 h to infinity (AUC_inf_) were determined after intravenous administration of MeP-loaded NPs to animals. All calculations were performed using the PKSolver add-in program for Microsoft Excel [114].

### 3.14. Statistical Analysis

The results were expressed as mean (±S.D.) unless otherwise specified. Statistical calculations were carried out using Design Expert 7.0 (Stat-Ease Inc., version 7.0; Design-Expert software; Minneapolis, MN, USA)), Data Acquisition Station (DAS) (MREL Group of Companies, version 2.0; Data Acquisition Station, Ontario, Canada). Statistical analysis was performed using Student’s *t*-test or ANOVA test. *p* < 0.05 was considered as significant.

## 4. Conclusions

PLGA-based delivery systems represent an object of great interest in modern medicine. The controlled substance release is an essential parameter that facilitates the improvement of substances’ therapeutic effect. However, NP characteristics, such as PS and LC, have to be optimized. In this study, we applied BBD to optimize the process of MeP-loaded NP formulation. Thorough formulation process optimization followed by scrupulous NPs characterization revealed good BBD applicability for the MeP-loaded NP design, especially in Mn- and Co-containing porphyrins. The release profiles of MnClTPP-NPs and CoTPP-NPs showed a sustained release of MeP from the polymer and dependence on the EE and binding constant of the MeP-PLGA complex. The preliminary acute cytotoxicity analysis demonstrated high safety of the formulated NPs. The plasma concentrations of MnClTPP-NPs and CoTPP-NPs were lower than those of free MeP during the distribution and elimination phases, indicating fast compound elimination from the bloodstream. Nevertheless, the encapsulation of NiTPP non-significantly affected the release profile and pharmacokinetic parameters of the formulation due to the predominant surface entrapping MeP demonstrating an initial strong release burst in vitro and a high NiTPP plasma concentration. These results demonstrated that MnClTPP-NPs and CoTPP-NPs were able to accumulate specifically in tissues, avoiding wide distribution via long-term circulation of the hydrophobic drug; in contrast, NiTPP-NPs demonstrated a high degree of initial release. The revealed differences in properties may promote the designed NPs’ application in totally different situations. We demonstrated the antitumor activity of MeP/MeP-NP combinations with AA and confirmed ROS generation when treated with a catalyst system. Eventually, we demonstrated the certain factors’ universality for the preparation of NPs loaded with tetrapyrrole macroheterocyclic compounds, BBD validity, and high potential of MnClTPP-NP, CoTPP-NP, and NiTPP-NP application in drug delivery and perspectives for further antitumor activity investigation.

## Figures and Tables

**Table 1:** Observed responses in BBD of NiTPP-loaded NPs (*X*_1_—PLGA, mg; *X*_2_—PVA, %; *X*_3_—organic to aqueous phase ratio; *Y*_1_—NiTPP LC, %; *Y*_2_—PS, nm).

No.	Independent Variable			Dependent Variable	
	*X*_1_	*X*_2_	*X*_3_	*Y*_1_	*Y*_2_
**1**	−1	0	−1	4.6 ± 0.2	395.0 ± 4.2
**2**	−1	0	1	13.5 ± 0.5	376.3 ± 9.7
**3**	0	0	0	3.1 ± 0.9	350.3 ± 6.8
**4**	1	0	1	2.9 ± 0.2	272.0 ± 4.1
**5**	−1	1	0	6.7 ± 0.4	372.1 ± 6.6
**6**	0	0	0	3.2 ± 0.3	370.2 ± 3.7
**7**	1	1	0	2.6 ± 0.4	453.5 ± 9.6
**8**	0	−1	1	5.3 ± 0.7	434.8 ± 11.3
**9**	0	0	0	3.0 ± 0.5	338.2 ± 5.8
**10**	1	0	−1	1.9 ± 0.1	557.1 ± 7.3
**11**	1	−1	0	4.8 ± 0.8	337.6 ± 4.9
**12**	0	0	0	2.5 ± 0.9	346.7 ± 8.3
**13**	0	0	0	3.3 ± 0.7	299.9 ± 2.2
**14**	−1	−1	0	19.9 ± 0.4	341.2 ± 5.5
**15**	0	1	−1	2.6 ± 0.5	418.7 ± 4.2
**16**	0	1	1	4.7 ± 0.8	408.7 ± 2.2
**17**	0	−1	−1	4.3 ± 0.7	700.4 ± 19.8

**Table 2:** Regression analysis and ANOVA of the NiTPP response surface models.

Models	R^2^	Adjusted R^2^	*F*-Value	*p*-Value Prob > F
**Response *Y*_1_ (NiTPP loading content, %)**				
*Linear*	0.5590	0.4572	215.08	<0.0001
2*FI*	0.6943	0.5109	223.43	<0.0001
*Quadratic*	0.9049	0.7827	138.07	0.0002
**Response *Y*_2_ (particle size, nm)**				
*Linear*	0.2887	0.1246	19.04	0.0061
2*FI*	0.5068	0.2110	19.60	0.0062
*Quadratic*	0.8258	0.6019	12.98	0.0158

**Table 3:** Comparison of the observed and predicted data for MeP-loaded NPs obtained using BBD.

MeP	Conditions	Responses	Observed Data	Predicted Data	Relative Error, %
**NiTPP-NPs**	*X*_1_: 5 mg *X*_2_: 1.47% *X*_3_: 1:7 *Desirability* 0.835	*Y*_1_, %	11.1	10.1	−9.9
		*Y*_2_, nm	322.9	296.9	−8.8
**CoTPP-NPs**	*X*_1_: 7.2 mg *X*_2_: 2% *X*_3_: 1:5 *Desirability* 0.750	*Y*_1_, %	8.7	10.3	15.2
		*Y*_2_, nm	344.5	410.5	16.1
**MnClTPP-NPs**	*X*_1_: 5 mg *X*_2_: 1.42% *X*_3_: 1:5.6 *Desirability* 0.837	*Y*_1_, %	28.9	22.6	−27.8
		*Y*_2_, nm	205.2	247.4	17.1

**Table 4:** MeP loading content (LC), encapsulation efficiency (EE), particle size (PS), zeta potential, and polydispersity index (PDI) of PLGA NPs containing NiTPP, CoTPP, and MnClTPP.

MeP	LC, %	EE, %	PS, nm	Zeta Potential, mV	PDI
**NiTPP-NPs**	11.1 ± 0.6	24.1 ± 0.9	322.9 ± 9.7	−14.7 ± 1.7	0.172
**CoTPP-NPs**	8.7 ± 0.4	79.7 ± 2.2	344.5 ± 15.6	−10.7 ± 2.3	0.191
**MnClTPP-NPs**	28.9 ± 1.6	79.9 ± 1.8	205.2 ± 10.2	+18.1 ± 1.6	0.140

Data are presented as mean ± S.D. (*n* = 3).

**Table 5:** Binding constants for PLGA-MeP complex in comparison with EE and LC data of NPs, containing the corresponding MeP, where *K_d_* is the binding constant.

MeP	NiTPP	CoTPP	MnClTPP
***K_d_***	0.2 × 10^−3^	0.2 × 10^−3^	10 × 10^−3^
**EE, %**	24.2	79.7	79.9
**LC, %**	11.1	8.7	28.9

**Table 6:** Hemolysis activity of MeP-loaded NPs and unloaded PLGA NPs.

Scheme	Concentration, mg/mL	Hemolysis, %
**blank NPs**	25	3.8
	2.5	1.4
	0.25	0.8
**NiTPP-NPs**	25	6.5
	2.5	2.2
	0.25	0.3
**CoTPP-NPs**	25	3.3
	2.5	1.3
	0.25	0.3
**MnClTPP-NPs**	25	107.5
	2.5	102.5
	0.25	88.1
	0.09	23.4
	0.009	1.8

**Table 7:** Model fitting of in vitro MeP release kinetics of optimum formulations.

MeP	Mathematical Model				
	Zero Order	First Order	Higuchi	Hixson-Crowell	Korsmeyer–Peppas
	*R* ^2^	*R* ^2^	*R* ^2^	*R* ^2^	*R* ^2^
**NiTPP-NPs**	−10.1967	0.7965	−7.4722	−8.4886	0.9948
**CoTPP-NPs**	−5.7916	0.6060	−3.5087	−4.2651	0.9876
**MnClTPP-NPs**	−2.8711	0.0180	−1.3010	−1.8479	0.9763

**Table 8:** Acute toxicity analysis (BALB/c female mice).

Dose, mg/kg	Formulation (Mice Survived)					
	*NiTPP*	*CoTPP*	*MnClTPP*	*NiTPP-NPs*	*CoTPP-NPs*	*MnClTPP-NPs*
**6.25**	3/3	3/3	3/3	-	-	-
**12.5**	3/3	3/3	3/3	-	-	-
**25**	1/3	2/3	3/3	-	-	-
**50**	0/3	0/3	2/3	-	-	-
**100**	-	-	-	3/3	3/3	3/3
**200**	-	-	-	3/3	3/3	3/3

**Table 9:** Plasma pharmacokinetic parameters of MeP and MeP-loaded NPs. Data are shown as mean ± S.D. (*n* = 3).

Parameters	Formulations						Units
	*MnClTPP*	*MnClTPP-NPs*	*CoTPP*	*CoTPP-NPs*	*NiTPP*	*NiTPP-NPs*	
**AUC_inf_**	15,362 ± 5377	9362 ± 3745 *	60,370 ± 18,111	1406 ± 211 *	612 ± 184	1133 ± 453	(µg/mL) * (h)
**Vd**	0.013 ± 0.004	0.188 ± 0.066 *	0.003 ± 0.001	3.083 ± 0.462 *	2.683 ± 0.805	2.614 ± 0.915	**L/kg**
**t1/2**	44 ± 14	37 ± 14 *	17 ± 4	33 ± 5 *	19 ± 6	24 ± 8	**h**
**MRT**	62 ± 21	53 ± 21 *	27 ± 7	66 ± 8 *	25 ± 6	45 ± 16	**h**

* *p* < 0.05 compared with free MeP.

**Table 10:** Variation factors and responses with levels for the preparation of MeP-based NPs.

Variation Factors		Levels	
		Low	High
***X*_1_**	**PLGA, mg**	5	45
***X*_2_**	**PVA, %**	0.5	2
***X*_3_**	**O/W (*v*/*v*)**	0.05	0.2

## Data Availability

Data is contained within the article.

## Supplementary Materials

The following are available online at https://www.mdpi.com/article/10.3390/ijms222212261/s1.
