# Supplementary material for: Optimization, Characterization and Pharmacokinetic Study of Meso-Tetraphenylporphyrin Metal Complex-Loaded PLGA Nanoparticles
Source: Int J Mol Sci. 2021 Nov 12;22(22):12261. doi: 10.3390/ijms222212261 (PMC8618356; doi:10.3390/ijms222212261)
Supplement: Supplementary file 1 [file ijms-22-12261-s001.zip › ijms-1434346-supplementary.pdf]

SUPPLEMENTARY MATERIALS

Optimization, characterization and pharmacokinetic study of meso-tetraphenylporphyrin metal complexes loaded PLGA nanoparticles

Mariia R. Mollaeva [1,2,*], Nikita Yabbarov [1,2], Maria Sokol [1,2], Margarita Chirkina [1,2], Murad D. Mollaev [2,3], Artur Zabolotskii [2,4], Irina Seregina [4], Mikhail Bolshov [4], Alexander Kaplun [5] and Elena Nikolskaya [1,2,*]

S1.1. *Optimization of Process Parameters for CoTPP-loaded NPs Preparation*

As a result of 17 experiments, nanoparticles with CoTPP loading content from 0.4% to 10.9% and average diameters ~ 203-714 nm were obtained (Table S1).

Table S1. Variation factors of the CoTPP-loaded nanoparticles formulation process and response functions.

№	Independent variable			Dependent variable	
	X_1	X_2	X_3	Y_1	Y_2
1	0	0	0	2.4±0.2	589.5±18.2
2	0	0	0	2.2±0.8	606.0±15.6
3	1	1	0	0.9±0.6	396.6±7.6
4	1	-1	0	1.3±0.8	319.2±4.1
5	-1	0	1	8.1±0.6	572.0±12.0
6	-1	-1	0	6.3±0.2	442.4±10.1
7	0	1	-1	0.4±0.1	206.1±1.3
8	-1	1	0	10.9±0.7	671.6±16.4
9	0	0	0	2.5±0.3	597.0±19.6
10	1	0	-1	2.1±0.5	685.9±14.1
11	0	1	1	2.2±0.1	203.4±2.0
12	0	0	0	2.4±0.2	587.1±17.8
13	0	-1	1	1.6±0.8	251.9±2.0
14	-1	0	-1	1.3±0.1	544.3±15.6
15	0	-1	-1	2.5±0.6	241.4±1.2
16	1	0	1	0.9±0.6	714.3±14.9
17	0	0	0	2.4±0.3	595.7±16.9

The adequacy of mathematical model was expressed by the determination coefficient R^2. The statistical significance was tested using Fisher's F-test (Table S2).

Table S2. Regression analysis and ANOVA for the CoTPP response surface models.

Models	R-Squared	Adjusted R-Squared	F-Value	p-value Prob > F
Response Y_1 (CoTPP loading content, %)				
Linear	0.5065	0.3927	491.16	<0.0001
2FI	0.6924	0.5079	458.97	<0.0001
Quadratic	0.8725	0.7086	379.65	<0.0001
Response Y_2 (particle size, nm)				
Linear	0.0165	-0.2105	1018.37	<0.0001
2FI	0.0279	-0.5554	1509.78	<0.0001
Quadratic	0.8468	0.6499	474.68	<0.0001

The analysis of variance in ANOVA has shown that the quadratic model is reliable and accurate regarding pure error. It can also be noted (data not shown) that the input parameters X_1, X_1^2 in the analysis of total content and X_2, X_2^2 in the analysis of particle size turned out to be the most significant.

Further, according to the results of analysis, regression equations were formulated. Based on the calculated coefficients, nonlinear quadratic equations (equation S1, S2) were obtained, making it possible to predict the shape of the response surface:

$Y_1 = 2.37 – 2.69X_1 + 0.33X_2 + 0.8X_3 – 1.21X_1X_2 – 1.98X_1X_3 + 0.67X_2X_3 + 1.95X_{12} + 0.53X_{22} – 1.22X_{32}$ **(S1)**

$Y_2 = 594.96 – 14.29X_1 + 27.85X_2 + 7.99X_3 – 37.95X_1X_2 + 0.17X_1X_3 – 3.30X_2X_3 + 132.96X_{12} – 270.47X_{22} – 98.79X_{32}$ **(S2)**

Based on quadratic equation (equation S1), it can be noted that PVA and the organic/aqueous (O/W) ratio are positively significant factors, while PLGA is negatively significant. Moreover, PVA and the O/W ratio interact positively, in contrast to PLGA, showing a negative interaction with PVA and the O/W ratio. Figure S1 (A,B) shows a strong effect of PLGA on the CoTPP loading content, while variation of PVA and the O/W ratio does not reveal any significant changes. It is known that the polymer weight influences the loading content of a substance and the average size of nanoparticles. Probably, due to a low PLGA mass in solution, the organic solvent diffuses faster into the aqueous phase to form nanoparticles [48,49].

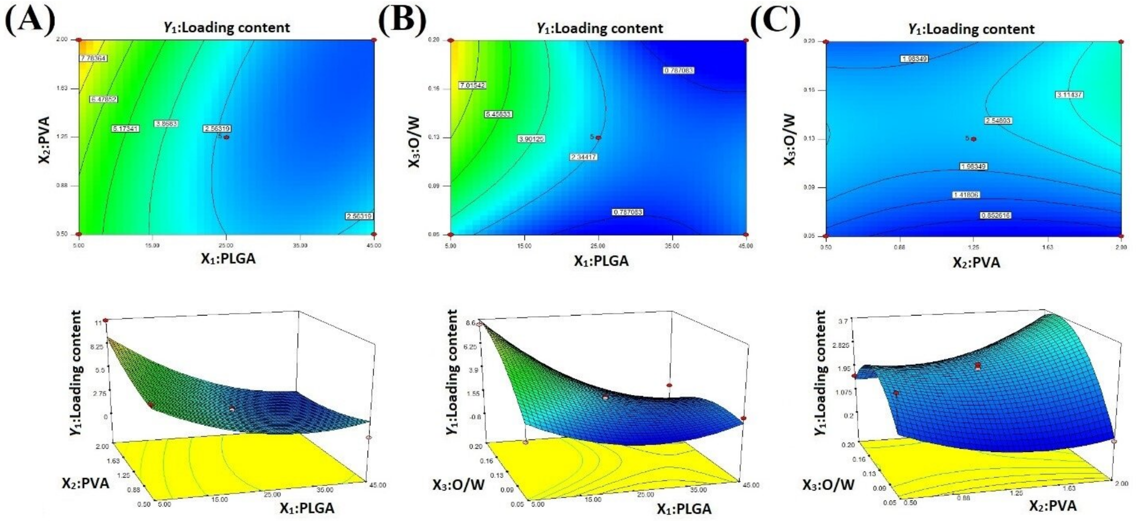

Figure S1. Two- and three-dimensional diagrams of the CoTPP loading content response surface depending on PLGA (A), PVA (B) and O/W ratio (C).

The quadratic equation for particle size response (equation S2), like the previous one, shows that PLGA is a negatively significant factor. However, the interaction PVA-PLGA and PVA-O/W ratio is negatively significant: neither the PLGA mass nor the O/W ratio possesses a significant effect on the particle size (Figure S2). However, with a decrease in the PVA concentration (to 0.5%), the particle size can reach ~ 180 nm. A low concentration of PVA reduces the viscosity of the aqueous phase and facilitates the diffusion of the organic solvent, leading to the formation of particles with a smaller size [51].

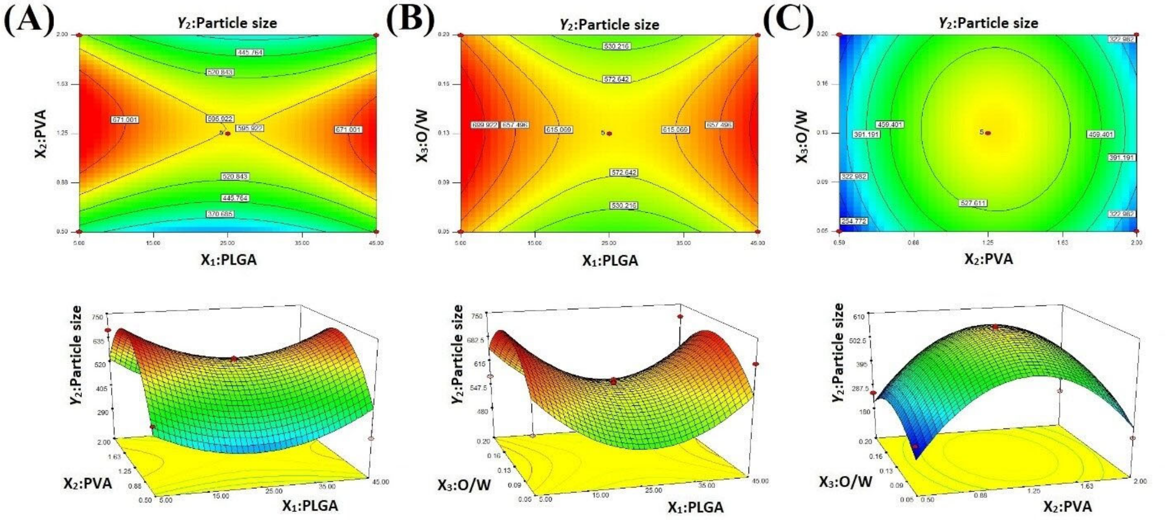

Figure S2. Two- and three-dimensional diagrams of the particle size response surface depending on PLGA (A), PVA (B) and O/W ratio (C).

Based on the specified characteristics, such as the minimized particle size and maximized CoTPP loading content, 22 options for optimizing nanoparticles were obtained. Among all the results, the factors values with the highest desirability (0.750) were selected and the best parameters were 7.22 mg of PLGA, 2% PVA and the O/W ratio = 1:5. The predicted value for CoTPP loading content is 10.3%, while that for the particle size – is 409.6 nm. Along with LC and PS, optimized NPs were analyzed for EE, zeta potential and PDI (Table 4).

S1.2. *Optimization of Process Parameters for MnClTPP-loaded NPs Preparation*

In order to optimize MnClTPP-loaded particles by Box-Behnken method, 17 experiments were carried out. The resulting particles possessed a MnClTPP total content from 3.4% to 17.0% and particle sizes 247.0 – 485.0 nm (Table S3).

Table S3. Variation factors of the MnClTPP-loaded nanoparticles formulation process and response functions

№	Independent variable			Dependent variable	
	X_1	X_2	X_3	Y_1	Y_2
1	0	0	0	10.1±0.5	257.3±3.9
2	0	0	0	10.2±0.6	249.5±3.7
3	1	1	0	5.7±0.2	287.0±1.9
4	1	-1	0	7.8±0.4	269.7±2.3
5	-1	0	1	7.7±0.7	324.5±5.3
6	-1	-1	0	17.0±0.8	288.5±6.9
7	0	1	-1	3.4±0.3	381.3±6.6
8	-1	1	0	12.5±0.2	260.4±3.1
9	0	0	0	10.3±0.6	259.8±3.4
10	1	0	-1	7.4±0.9	357.5±5.3
11	0	1	1	10.3±0.4	303.5±4.8
12	0	0	0	10.2±0.5	247.4±2.5

13	0	-1	1	15.8±0.5	477.4±6.4
14	-1	0	-1	7.7±0.4	324.5±3.5
15	0	-1	-1	14.6±0.2	484.3±8.5
16	1	0	1	8.6±0.6	256.2±7.6
17	0	0	0	10.1±0.4	255.6±0.9

Using the determination coefficient R^2, there was a confirmation of the mathematical model adequacy. The statistical significance was tested using Fisher's F-test (Table S4).

Table S4. Regression analysis and ANOVA for the MnClTPP response surface models.

**Models**	**R-Squared**	**Adjusted R-Squared**	**F-Value**	**p-value Prob > F**
Response Y_1 (MnClTPP loading content, %)				
Linear	0.6454	0.5636	1848.64	<0.0001
2FI	0.8639	0.7822	1063.97	<0.0001
Quadratic	0.9142	0.8038	1341.31	<0.0001
Response Y_2 (particle size, nm)				
Linear	0.1800	-0.0092	295.54	<0.0001
2FI	0.2136	-0.2582	425.13	<0.0001
Quadratic	0.8972	0.7649	110.03	0.0003

Taking into account the ANOVA results, it can be concluded that the 2FI model is reliable for the MnClTPP loading content, while the quadratic model is reliable and accurate regarding pure error in the case of the particle size. The most significant parameters in the analysis of the MnClTPP loading content were the input parameters X_1 and X_3 (data not shown) and parameters X_2, X_2^2 in the case of particle size analysis.

The linear model for the MnClTPP loading content and the quadratic model for the particle size were expressed by equations S3 and S4 as given below:

$Y_1 = 9.31 + 0.16X_1 – 8.10X_2 + 113.04X_3 + 0.04X_1X_2 – 3.65X_1X_3 + 25.78X_2X_3$ **(S3)**

$Y_2 = 670.78 + 5.62X_1 – 307.66X_2 – 3879.86X_3 + 0.76X_1X_2 – 11.78X_1X_3 – 315.11X_2X_3 – 0.10X_1^2 + 112.07X_2^2 + 16829.33X_3^2$ **(S4)**

The polynomial regression equation (equation S3) indicated that X_2 has a negative effect on the loading content, while X_1 and X_3 were favorable factors. Perhaps a positive effect of these factors is associated with the complexity of MnClTPP release from a polymer matrix with a high viscosity. A decrease in the aqueous phase contributes to a slow diffusion of chloroform into water, hence a greater amount of MnClTPP remains inside the nanoparticles [48].

Moreover, Figure S3 (A,C) demonstrated a strong effect of X_2 on MnClTPP loading content, while variation of X_1 and X_3 did not reveal any significant changes. A low PVA concentration may contribute to a high MnClTPP loading content due to a decrease in the surface tension of the primary emulsion [49].

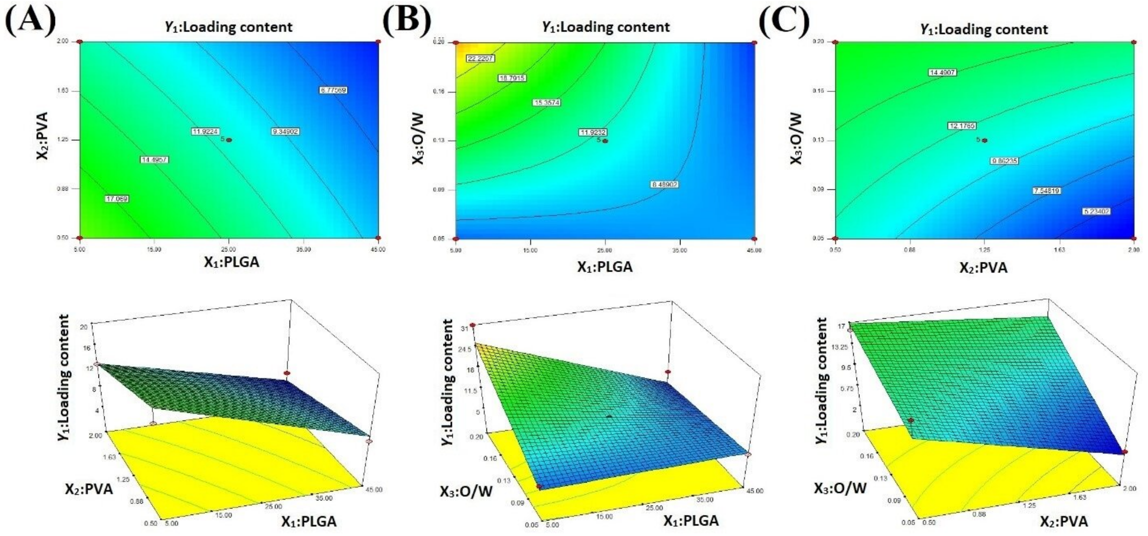

Figure S3. Two- and three-dimensional diagrams of the MnClTPP loading content response surface depending on PLGA (A), PVA (B) and O/W ratio (C).

As can be seen in the particle size response quadratic equation (equation S4), X_1 is a negatively significant factor which indicated that an increase in the PLGA mass decreases the particle size. However, as shown in Figure S4, X_1 did not possess a significant effect on the particle size. Meanwhile, the interaction of X_3 with X_1 and X_2 is negatively significant. Figure S4 (C) clearly demonstrates a ''canopy type'' of the response surface, indicating an initial nonlinear decrease accompanied by an increase in the MnClTPP-loaded particle size. Perhaps, a positive interaction between X_2 and X_3 factors affects the viscosity of the solution: a viscosity decrease induced by a decrease in the PVA concentration or an increase in the volume of the water phase may lead to a loss of particle stabilization and aggregation [52].

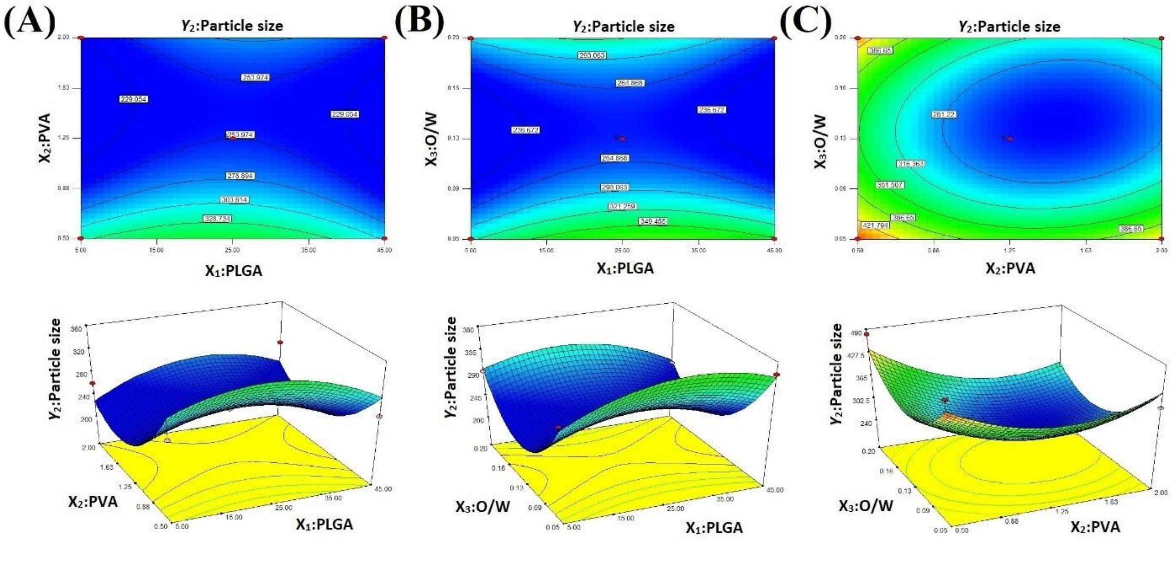

Figure S4. Two- and three-dimensional diagrams of the particle size response surface depending on PLGA (A), PVA (B) and O/W ratio (C).

Thus, we received 13 solutions for optimizing NPs using the Box-Behnken experimental design. To obtain NPs with a minimized particle size and a maximized MnClTPP loading content, the factors values with the highest desirability (0.837) were selected: 5 mg of PLGA, 1.42% PVA

and the O/W ratio = 1:5.56. The predicted value for the MnClTPP loading content is 22.6%, while for the particle size 247.4 nm. Along with LC and PS, optimized NPs were analyzed for EE, zeta potential and PDI (Table 4).

S2.1. *In Vitro Cytotoxicity of the ascorbic acid*

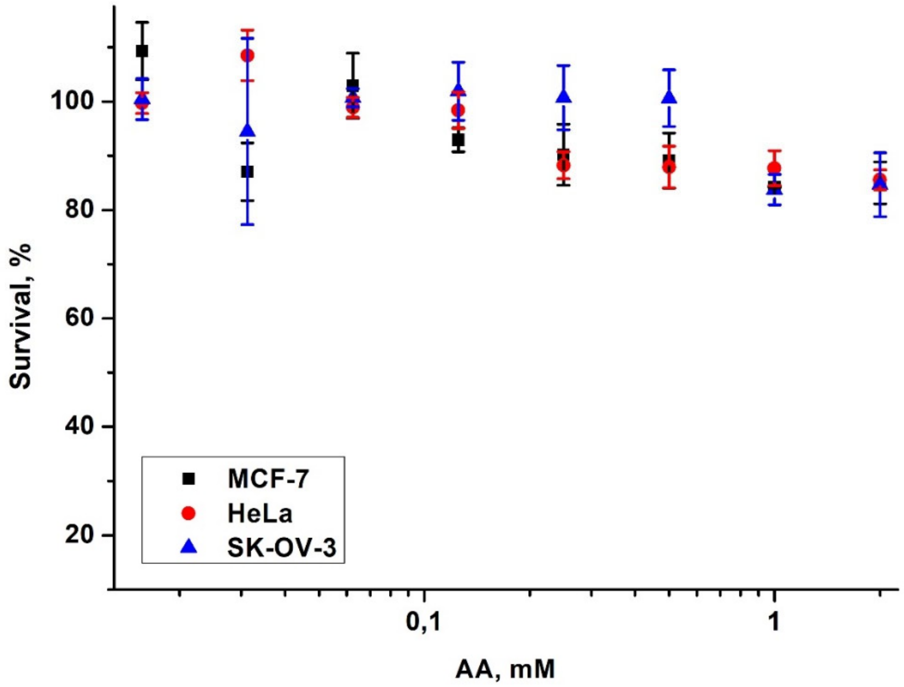

Figure S5. Cytotoxic activity of the ascorbic acid (AA) against MCF7, HeLa and SK-OV-3 cells after 72 h of incubation.
